# Association of Melatonin Production with Seasonal Changes, Low Temperature, and Immuno-Responses in Hamsters

**DOI:** 10.3390/molecules23030703

**Published:** 2018-03-20

**Authors:** Xiaoying Xu, Xiaoyan Liu, Shuran Ma, Ya Xu, Ying Xu, Xiazhen Guo, Dekui Li

**Affiliations:** 1 Beijing University of Chinese Medicine, Beijing 100029, China; xuxy@bucm.edu.cn (X.X.); liuxy1088@sina.com (X.L.); mashuran64@sina.com (S.M.); xuya@bucm.edu.cn (Y.X.); guoxiazhen@126.com (X.G.); 2 World Federation of Chinese Medicine Societies, Beijing 100101, China; 7384321@163.com

**Keywords:** melatonin, hamster, immunity response, *AANAT*, *ASMT*, cold weather

## Abstract

Seasonal changes impact the melatonin production and immuno-activities in vertebrates. This is believed due to the photoperiodic alterations of the different seasons which impact the functions of pineal gland. The short photoperiod promotes pineal melatonin production. As a result, during the winter, animals have significantly higher levels of melatonin than in summer. However, the seasonal changes also include temperature changes. This factor has never been systemically investigated in animals. In the current study, we observed that increased temperature had limited influence on melatonin production. In contrast, cold temperature is the major factor to induce melatonin production in hamsters. Cold temperature per se can upregulate the expressions of melatonin synthetic gene *AANAT* and *ASMT*, which are the important enzymes for melatonin biosynthesis. The elevated melatonin levels induced by the cold exposure in hamster in turn, improve the immuno-responses of the animals with increased levels of IL1, 6, and 10 as well CD3. In addition, melatonin as a potent antioxidant and thermogenic agent would improve the survival chance of animals during cold weather.

## 1. Introduction

Melatonin is a signaling molecule to reflect environmental photoperiodic alterations in vertebrates [1] and also in other low rank of organisms including zooplankton [2]. Its production is usually associated with darkness, and therefore, it is referred to as a chemical expression of darkness [3]. Many organs, tissues, and cells were proved to synthesize melatonin [4]. However, the pineal gland is currently the only organ responsible for the melatonin circadian rhythm. This rhythm is important for the chronobiologic activities including sleep, seasonal reproduction, jet lag, and especially the immuno-responses in mammals. For example, immune-activity exhibits seasonal variations, and these variations may be partially related to melatonin levels [5]. Melatonin is a strong immune regulator. It significantly increases the tolerance of organisms to biological stress. For example, melatonin treatment significantly reduced the death rate of mice infected by *Schistosoma mansoni* [6]. It suppressed the inflammatory reactions and upregulated the cell immuno-response in hamsters infected by human liver fluke [7]. It was hypothesized that melatonin downregulated the inflammatory activity and upregulated the specific immuno-responses [8]. This effect was even observed in plants. Melatonin treatment significantly increases the resistance of plants against the bacteria and fungi [9,10,11].

For decades, the regulatory mechanisms of melatonin synthesis in the pineal gland were focused on the impact of light exposure or photoperiodic changes. Definitely, light and photoperiod changes are the major regulators of melatonin production in pineal gland of vertebrates [12]. However, what is the influence of temperature, particularly the cold temperature on melatonin production in animals is virtually unknown. Temperature as an important factor of the seasonal changes in addition to the photoperiod should be given a consideration regarding its effects on melatonin synthesis and its potential biological consequences. In the current study, the effects of climate changes including photoperiod and temperature on melatonin production in hamster are systemically investigated. Particularly, the influence of cold temperature on melatonin production and its association on immuno-responses in these animals are also studied.

## 2. Results

### 2.1. Effects of Seasonal Changes on the Gene Expression of AANAT and SAMT in Pineal Glands of Hamsters As Well As on Their Serum Melatonin Levels

**Figure 1:**
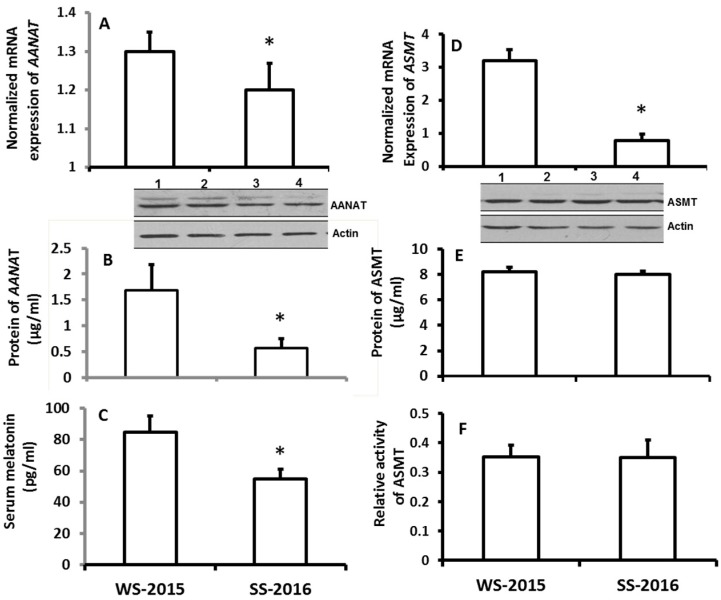
The effects of naturally seasonal alterations on the expression of melatonin synthetic genes, *AANAT* and *ASMT*, in the pineal gland and on melatonin production in hamsters. The animals were raised on the natural climate conditions (local environmental temperature, photoperiod and humidity) for one months and these animals were sacrificed at 9–10 pm on the days of winter solstice 2015 (WS-2015) or summer solstice 2016 (SS-2016), respectively. Pineal glands were collected for melatonin synthetic gene expression assay and serum was collected for melatonin assay. Animals were sacrificed under dim red light. The inserts were the images of Western Blots of the proteins of *AANAT* and *ASMT*, respectively. In panel **B**, the *AANAT* proteins of lines 1 and 2 were from the samples of WS-2015, line 3 and 4 were from SS-2016. In panel **E**, the *ASMT* proteins of lines 1 and 2 were from the samples of WS-2015, line 3 and 4 were from SS-2016. **A** and **B**: mRNA and protein levels of *AANAT*, respectively; **C**: Serum melatonin level; **D**, **E** and **F**: mRNA, protein levels and activity of *ASMT*, respectively. The data were expressed as means ± SDs of 16 hamsters each group. The gene expressions were normalized against housekeeping gene actin and the changes was analyzed with the 2^−△△Ct^ method. * Significant difference vs. WS-2015.

To investigate the potential association regarding the seasonal changes and the alteration of melatonin production, hamsters were selected due to their sensitivity to the changes of the photoperiod. Also, summer and winter were selected because they have the most distinguishable climates. In the first study, the animals were exposed to the natural climates of both seasons. It was found that the gene expressions of *AANAT* including their mRNAs and proteins in the pineal gland of hamsters were significantly upregulated in the winter compared the summer (Figure 1A,B).

**Figure 2:**
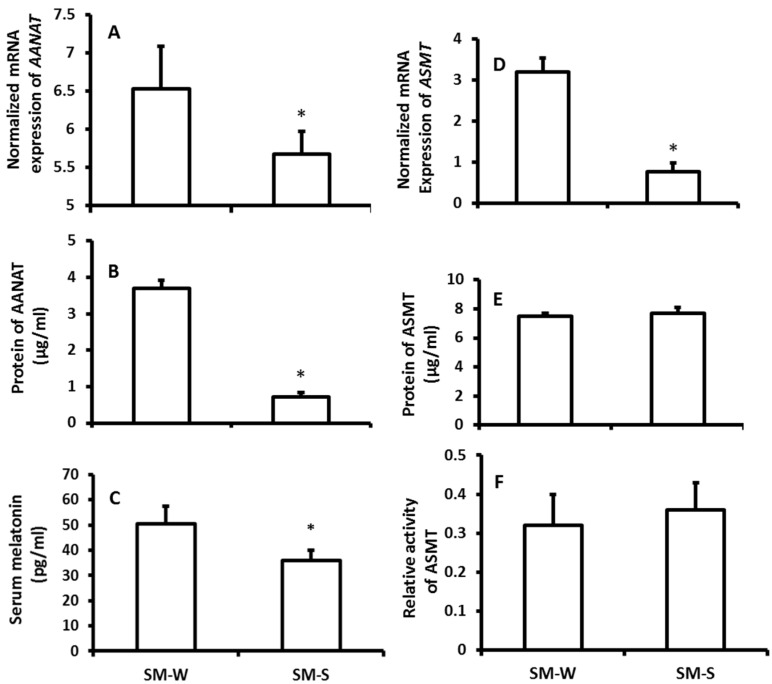
The effects of simulated-seasonal changes on the expressions of melatonin synthetic genes, *AANAT* and *ASMT*, in pineal gland and serum melatonin levels of hamsters. The animals were raised in the seasonal-simulated system for one month. The system simulated the local winter of 2015 (SM-W) and summer of 2016 (SM-S) climate changes including the temperature, photoperiod and humidity. The animals were sacrificed at 9–10 pm on the days of simulated winter solstice and summer solstice, respectively. Pineal glands were collected for melatonin synthetic gene expression assay, and serum was collected for melatonin assay. Animals were sacrificed under dim red light. **A** and **B**: mRNA and protein levels of *AANAT*, respectively; **C**: Serum melatonin level; **D**, **E** and **F**: mRNA, protein levels and activity of *ASMT*, respectively.The data were expressed as means ± SDs of 16 hamsters each group. The gene expressions were normalized against housekeeping gene actin and the changes was analyzed with the 2^−△△Ct^ method. * Significant difference vs. SM-S.

The mRNA expression of *ASMT* of pineal gland was significantly upregulated in the wither group compared to the summer group; however, the protein level of *ASMT* did not significantly altered between the seasons (Figure 1D,E). Thus, the ASMT activity was performed to confirm this observation. It was in accordance with its protein levels its activity was also exhibited no significant difference between winter and summer (Figure 1F). The results showed that the serum melatonin levels of the animals were significantly higher in winter than that in summer (Figure 1C). To confirm these observations, in the second study, rather than exposing them to the natural climate, the animals were housed in the seasonal-simulated system for a month. This system was controlled via computer and accurately mimicked the climate changes of summer and winter, respectively, including the temperature, photoperiod, and humidity. The results indicated that the gene expression of melatonin synthetic enzymes as well as the melatonin production exhibited similar patterns to the animals that were exposed to natural climate alterations (Figure 2).

### 2.2. The Influence of the Seasonal Changes on the Indexes of Immuno-Responses

**Figure 3:**
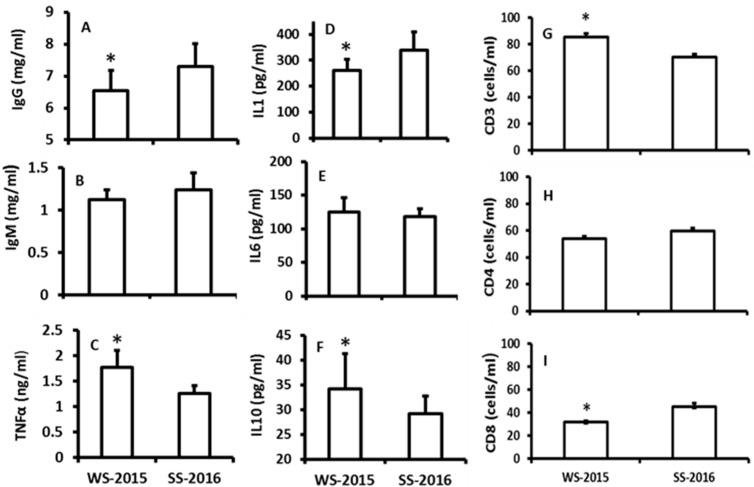
The effects of naturally seasonal alterations on the immuno-responses of hamsters. WS-2015: winter solstice of 2015; SS-2016: summer solstice of 2016. **A**, **B** and **C**: the levels of IgG, IgM and TNFα, respectively; **D**, **E** and **F**: the levels of IL1, 6 and 10, respectively; **G**, **H** and **I**: the numbers of CD3, 4 and 8, respectively. The data were expressed as means ± SDs of 16 hamsters each group. * Significant difference vs. SS-2016.

The effects of seasonal changes on the immuno-indexes including TNF-α, IgG, IgM, IL-1, IL-6, IL-10, CD3, CD4, and CD8 cells were investigated. The data indicated that there were no significant differences among IgM, IL-6 and CD4+ between the seasons (Figure 3B,E,H). The levels of TNF-α, IL-10, and CD3+ were significantly increased in the winter animals when they were compared to the summer animals (Figure 3C,F,G). In contrast, the levels of IgG, IL-1, and CD8+ were significantly lower in the winter animals than those of summer animals (Figure 3C,F,G).

### 2.3. The Effects of Temperature Changes on Melatonin Production and the Indexes of Immuno-Responses

**Figure 4:**
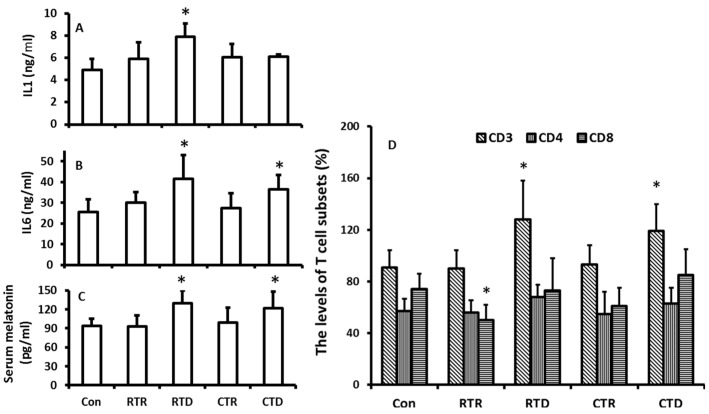
Temperature changes on the melatonin production and immuno-responses of hamsters. The animals were housed in the fixed ligh/dark cycle (12/12 h) and humidity but different temperature patterns. Con: 22 ± 2 °C; RTR: rapid temperature rising; RTD: rapid temperature drop; CTR: constant temperature rising and CTD: constant temperature drop, respectively. Animal were sacrificed after 72 h of the treatments at 9–10:00 pm under dim red light. Serum was collected for the further assays. **A** and **B**: the levels of IL1 and 6, respectively; **C**: the serum melatonin level, **D**: the distributions of CD3, 4 and 8 in different treatment groups. The data were expressed as means ± SDs of eight hamsters each group. * Significant difference vs. controls.

In this experiment, the temperature was isolated from other factors of the climate including photoperiod and humidity. The effects of temperature on melatonin production and immuno-indexes in the hamsters were evaluated. The results showed that the rising temperature appeared to have no significant impact on the melatonin production in the hamsters (Figure 4C); however, the temperature decline including the rapid decline and constant low temperature played major role as to promote the melatonin production (Figure 4C). The temperature decline also influenced the immuno-indexes of the animals. For example, the levels of IL1, IL6, and CD3+ were significantly higher in low temperature treated groups than those in the control. A high temperature treatment did not significantly modify these indexes (Figure 4A,B,D). The results indicated that the increased levels of IL1, IL6, and CD3+ were positively related to the melatonin levels.

## 3. Discussions

It is well documented that melatonin levels in vertebrates exhibit circadian rhythms with high level at darkness and low baseline during the day. This circadian rhythm is regulated by the alterations of photoperiods via suprachiasmatic nucleus (SCN) [13]. It is logically believed that melatonin secretory peak should be much longer in winter than that in other seasons due to its short photoperiod of this season. The prolonged melatonin secretory peak was reported in different species during the winter compared to other seasons [14,15]. It is obvious that the climate changes during winter have a short photoperiod and lower temperature. To adapt to these changes, organisms have to alter their physiological activities and patterns of their metabolisms. Melatonin is the major signaling to transduce these environmental changes to the organisms. In vertebrates, this signaling melatonin is generated by the pineal gland rather than the extra-pineal melatonin [4,16]. In the past, concern for the regulation of the melatonin signal was given to the photoperiod changes, and little focus was given to the influence of cold weather on melatonin production. A study only reported that the combination of photoperiod and temperature had the effect on melatonin production in trout [17]. In the current study, in addition to systemically investigating the seasonal changes on melatonin production, the temperature was isolated from the climate changes, and its effect on melatonin production was, for first time, thoroughly explored in hamsters. The hamster is a sensitive animal model to study the photoperiodic alterations as well as temperature changes [18]. In our first study, the animals were exposed to the natural climate for one month, and the termination dates of the study were selected at either the summer solstice, which is the longest photoperiodic day, or winter solstice, which is shortest one of the year, respectively. The results revealed that the gene expressions of *AANAT* including the levels of mRNA and protein were significantly upregulated in the pineal gland of hamsters in winter solstice compared to the summer solstice. However, it is more complicated for *ASMT*. The mRNA expression of *ASMT* was significantly upregulated in the winter compared to the summer. Unexpectedly, the protein level of the *ASMT* as well as the enzyme activity exhibited no significant differences with summer animals. This is probably related to the posttranslational modification of the *ASMT*. In consistent with other reports, melatonin production is significantly higher in the winter animals than that in summer animals. These observations can easily result in a conclusion that the *AANAT* rather than the *ASMT* is the rate-limited enzyme of melatonin synthesis in the pineal gland [19]. The question is why the *ASMT* expression would increase during winter if it was not associated with elevated melatonin production? There is a possibility that the proteins of *AANAT* and *ASMT* have different turnover rates, respectively, and so did their enzyme activities. Since the samples were collected only at one time point due to limited animal numbers, there is a chance that a potential high protein expression level of *ASMT* would be missed. To solve this problem, multi-times of sample collection are required. This is our future approach to continuing this study. Based on the data from the current study, it is difficult to exclude the contribution of *ASMT* on elevated melatonin levels in winter. Another alternative explanation on this phenomenon might be that the *AANAT* probably catalyzed the last step of melatonin formation rather than *ASMT* [20].

We considered that many unpredicted factors might interfere the study results when the animals were exposed to the natural climate. To further conform the results, the animals were raised in the climate simulated system including photoperiod, temperature, and humidity, which were well controlled by computers. Similar results regarding the melatonin synthetic gene expression and melatonin production were obtained as in the animals those were exposed to the natural climate. Thus, the results were confirmed. 

As mentioned previously, the seasonal changes include photoperiod as well as temperature. To the best of our knowledge, the influence of temperature on melatonin synthetic ability has not been investigated or has not been isolated from the photoperiodic alterations in any animal model. To explore the potential influence of temperature on melatonin production, the hamsters were housed under the same photoperiodic condition (12:12 h light:dark cycle) and fixed humidity (45–50%) but experienced different temperature alterations. In this approach, the only variable factor in different groups is the temperature. It was assumed that all alterations on melatonin production and related immunoresponses among the groups probably were caused by the temperature manipulations per se.

The results showed that a rapid increase in temperature or constant high temperature exposure did not significantly altered the melatonin production in the hamsters compared to their controls. In contrast, the rapid decline in temperature or constant low temperature exposure indeed significantly elevated the melatonin production of the animals compared to their controls. Thus, in addition to photoperiod (or light exposure), cold temperature is also a major factor to regulate the melatonin production. This factor has been ignored for decades. Recently, it was frequently reported that cold stress significantly upregulated gene expression of melatonin synthetic enzymes and melatonin productions in plants or microorganisms [21,22]. This increase promotes their tolerance to cold temperature and increases their survival chances during harsh environments due to the potent antioxidant property of melatonin [23,24,25]. The effect of cold temperature on melatonin production in animals was not available in the literature. Herein, we report that cold temperature induced melatonin production in hamsters, and the mechanism is upregulation of the gene expressions both of *AANAT* and *ASMT* in the pineal gland. This is important for animals to adapt the harsh cold weather. Animals are required to modify predominantly their endocrine and immune systems under extreme climatic events (for example, in the rapid drop in temperature) [26]. Increased melatonin production might be one of the strategies as observed in the study (Figure 3). Melatonin is a potent antioxidant [27] and is also an immuno-enhancement agent [28,29,30]. Melatonin production in the pineal gland significantly impacts the immuno-activities of animals against variety stressors. These include the sepsis, viruses, and parasitic infections [31,32,33]. The immuno-pineal axis and its potential mechanisms has been hypothesized to explain the association of melatonin and immunological activities [34]. Cold temperature could dampe the immuno-responses of animals [35], and the increased melatonin production could compensate this adverse effect of cold to elevate production of IgG and CD3+ (Figure 4). In addition, under cold temperatures, melatonin promotes the recruitment of the brown adipose tissue [36], which increases the thermogenesis in the animals during winter [37]. This is crucial for hibernating animals such as hamsters to survive during cold weather. In summary, we confirmed that seasonal changes modified the melatonin productions and that melatonin level was positively associated with the immuno-functions in animals. For the first time, we identified that, in addition to the photoperiodic information, cold temperature is a major factor to induce melatonin production. Low temperature upregulated the gene expression of *AANAT* and *ASMT* in the pineal gland; it therefore promoted the melatonin production and immuno-functions in hamster during winter.

## 4. Materials and Methods

### 4.1. Chemicals

Melatonin and all other reagents were purchased from Sigma-Aldrich (St. Louis, MO, USA); otherwise, the vendors were identified.

### 4.2. Animals

Two-month-old male Golden hamsters (*Mesocricetus auratus*) were purchased from Vital River Laboratories Co. Ltd. (Beijing, China), strain (LVG), genetic purity degree (SPF/VAF), certified number:SCXK(Jing)2012-0001. The animals were housed at a temperature of 22 ± 2 °C under a natural photoperiod and had access to food and water *ad libitum*. All experimental procedures were approved by the Animal Care Committee of the Beijing Traditional Medical University (Protocal No.: BUCM-4-2015092007-3007). After a week of acclimation, those hamsters were randomized for the following experiments.

### 4.3. Animal Studies

#### 4.3.1. Study of Natural Seasonal Changes on the Expression of Melatonin Synthetic Genes Including AANAT and ASMT in the Pineal Gland and Circulating Melatonin Production

Animals were divided into three groups with 16 hamsters in each group. These included control, summer, and winter groups, respectively. In control group, the animals were exposed to 12:12 h light/dark cycle under the room temperature 22 ± 2 °C and humidity 50% for one month. In the winter group, the animals were exposed to the winter natural photoperiod with the Beijing local winter temperature (average room temperature was around 15 °C). After one month of treatment, the animals were sacrificed on the day of the winter solstice (22 December 2015). In the summer group, the animals were exposed to the summer natural photoperiod with the Beijing local summer temperature (average room temperature was around 27 °C). After one month of treatment, the animals were sacrificed on the day of the summer solstice (22 June 2016). All animals were sacrificed at 9–10 pm under dim red light (<5 w). The animal study procedure is summarized in Table 1. The pineal glands were collected and stored at −80 °C for future analysis. Blood was collected, sitting on ice overnight, and the serum was stored at −80 °C for melatonin and other assays.

#### 4.3.2. Study of Simulated Summer and Winter Climate Changes on the Expression of Melatonin-Synthetic Genes of the Pineal Gland and Melatonin Production

Animals were divided into three groups with 16 hamsters in each group. These included control, summer, and winter mimic groups, respectively. In the control group, the animals were exposed to 12:12 h light/dark cycle under a room temperature of 22 ± 2 °C and humidity 45–50% for one month. In simulated summer group, the animals were housed on the computer-controlled summer simulated system for one month. The temperature was controlled at 27 ± 2 °C (this was the average summer room temperature of Beijing locally), humidity 45–50%, and the photoperiod was simulated to the local alterations by computer. In the winter mimic group, the animals were housed in the computer-controlled winter simulated system for one month. The temperature was controlled at 15 ± 2 °C (this was the average winter room temperature of Beijing locally), humidity was 45–50%, and the photoperiod was synchronized to the local alterations by computer. After one month of treatment, the animals were sacrificed at 9–10:00 pm. under dim red light (<5 °C). The pineal glands were collected and stored at −80 °C for future analyses. Blood was collected, sitting on the ice overnight, and the serum was stored in −80 °C for melatonin assay.

#### 4.3.3. Study of Extreme temperature Alterations on Melatonin Production and Immune-Response of Hamsters

Animals were divided into five groups with eight animals in each group. These included control (Col), rapid temperature rising (RTR), constant high temperature (CHT), rapid temperature decline (RTD), and constant low temperature (CLT) groups, respectively. All groups were exposed to the same natural photoperiod. In Col, the animals were under the controlled temperature 22 ± 2 °C. In RTR (*n* = 16), and the temperature was increased from 22 °C to 38 °C during 30 min and last for 6. In CHT, the animals were maintained at 35 °C for 72 h. In TRD, the temperature was reduced from 22 °C to 4 °C over 30 min and lasted for 6 h. In CLT, the temperature was maintained at 4 °C for 72 h. All animals were sacrificed after 72 h at 9–10 pm under dim red light (<5 w), and the serum was collected for future analyses. The animal study procedure is summarized in the Table 2.

### 4.4. Methods

#### 4.4.1. Melatonin Assay

Melatonin extraction: 600 µL of serum were mixed with 3 mL chloroform, vortexed, and then centrifuged at 8000 rpm for 30 min under 4 °C. The chloroform phase was collected and evaporated with vacuum. The extract was reconstituted with phosphate buff for melatonin assay. ^125^I radioimmunoassays (RIAs) was used for melatonin assay. The procedures were followed the instructions of the Melatonin Research RIA Kit (LDN GmbH & Co., KG, Nordhorn, Germany).

#### 4.4.2. Measurement of mRNA Expression of *AANAT* and *ASMT* in the Pineal Glands

Single pineal gland was homogenized with 100 µL phosphate buff and then centrifuged at 10,000× *g* for 15 min, under 4 °C. Total RNA was extracted using the TRIzol reagent (Invitrogen, Carlsbad, CA, USA) and immediately reverse-transcribed using Prime Script™ RT reagent Kit with g DNA Eraser (Ta Ka Ra). The RT-PCR reactions consisted of 10 μL Light Cycler^®^ Multiplex Masters (Roche Molecular Systems, Inc., Pleasanton, CA, USA, 25 μmol/L forward and reverse primers, 2 μL template, and dd H_2_O were added up to a total volume of 20 μL. The procedure was as follows: 95 °C for 10 min, 40 cycles of 95 °C for 10 s, and 60 °C for 10 s, melting curve from 65 °C to 95 °C, increasing in increments of 0.5 °C every 5 s. Normalization was performed using the housekeeping gene actin as a control. Relative mRNA expression was calculated by the 2^−△△Ct^ method. The primers for RT-PCR were listed in Table 3.

#### 4.4.3. Analysis of Protein Levels of *AANAT* and *ASMT* of the Pineal Glans

Total proteins were extracted by RIPA lysis buffer (Huaxingbio, Beijing, China) and boiled for 10 min. For SDS-PAGE, 10 μg of protein was loaded on 12% (*w*/*v*) SDS-PAGE gel, separated, and transferred onto PVDF membrane (Millipore, Bedford, MA, USA) using a semi-dry Trans-Blot apparatus (Bio-Rad, Hercules, CA, USA). As primary antibodies against β-actin, *AANAT*, and *ASMT*, rabbit Ab from Abcam (Cambridge, UK) were used (1/300 dilution). The secondary antibody was anti-mouse Ig G conjugated with Pro-light HRP Chemiluminescence detection reagents A and B (Tiangen Biotech, Beijing, Chuna) according to the manufacturer’s instructions.

#### 4.4.4. Measurement of the Activity of *ASMT*

The activity of *ASMT* was measured by enzyme-linked immunosorbent assay (ELISA) using the Hamster HIOMT ELISA Kit (RGB& CHN Lot: 02/2013). The measurement was followed by the instructions provided by the manufacturer. Simply, the 50 µL homogenate of a pineal gland was incubated with 40 µL of florescence labelled substrates of *ASMT* at 37 °C for 60 min. Then, 10 µL of stop solution was added to the mixture to terminate the enzyme reaction. The florescence change was detected to quantity the *ASMT* activity of a pineal gland. The unit was expressed as the relative density of the florescence detected in a pineal gland.

#### 4.4.5. Measurements of TNF-α, IL-1, IL-6, IgG, and IgM

TNF-α, IL-1, and IL-6 were examined by enzyme-linked immunosorbent assay (ELISA). The serum samples were added into TNF-α, IL-1 or IL-6 pre-coated wells, respectively. Then, the anti-hamster (TNF-α, IL-1 or IL-6) monoclonal antibodies were added, respectively. These antibodies were labeled with biotin and combined with Streptavidin-HRP to form an immune complex. The mixtures were incubated for 60 min at 37 °C and washed five times with phosphate buffer to remove the uncombined enzyme. After that, Chromogen Solution A and B was added into the sample separately. The reaction time was set up for 10 min at 37 °C. Then, a stop solution was added to terminate the reaction. The values of TNF-α, IL-1, or IL-6 were measured depending their OD intensity. All procedures were followed the manufacturer’s instructions. IgG and IgM were analyzed using IgG/M Assay (Kit RGB& CHN Lot: 201504018. 30013), Beckmancoulter UniCel DxC 800 Synchro (Brea, CA, USA) and followed the manufacturer’s instructions.

#### 4.4.6. Detection of T Lymphocyte Subsets (CD3, CD4, and CD8)

A double-antibody sandwich enzyme-linked immunosorbent assay (ELISA) was used to assay the levels of cluster of differentiations of CD3, CD4 and CD8, respectively. Simply, 40 μL blood samples were mixed with both anti-hamster T cells monoclonal antibodies and Streptavidin-HRP (each with 10 μL) and gently shaken and incubated 60 min at 37 °C. After washing to remove the membrane from the chromogen solution A (50 μL), then chromogen solution B (50 μL) were added, gently mixed, and incubated for 15 min at 37 °C to avoid light exposure. Then, 50 μL stop solutions were added to terminate the reaction (the blue mixture immediately changed to yellow). The samples were measure under 450 nm wavelength, and the OD values of the sample were recorded to calculate the clusters of differentiation of CD3, CD4, and CD8, respectively. 

### 4.5. Statistical Analyses

The data were expressed as the mean ± SD (*N* = 8–16) and analyzed using variance analysis of variance (ANOVA) followed by student *t* test using SPSS21.0 statistical software (SPSS, Inc., Chicago, IL, USA). *p* < 0.05 is considered statistically significant and *p* < 0.01 is considered statistically highly significant.

## Figures and Tables

**Table 1:** Summarization of the animal study procedure for 4.3.1.

Groups	Animals	Beginning Date	Treatments	Termination Date
Control	16	22 November 2015	12:12 h light/dark, 22 ± 2 °C, humidity (45–50%)	22 December 2015
Winter	16	22 November 2015	Local natural winter climate	22 December 2015
Summer	16	22 May 2016	Local natural summer climate	22 June 2016

**Table 2:** Summarization of the animal study procedure for 4.3.3.

Groups	Animals	Treatments	Duration	Termination
Col	8	22 °C.	72 h	9–10 pm under red light (<5 w)
RTR	8	22 rising to 38 °C during 30 min, last for 6 h, back to 25 °C	72 h	9–10 pm under red light (<5 w)
CHT	8	38 °C	72 h	9–10 pm under red light (<5 w)
TRD	8	22 declining to 4 °C during 30 min, last for 6 h, back to 25 °C.	72 h	9–10 pm under red light (<5 w)
CLT	8	4 °C	72 h	9–10 pm under red light (<5 w)

Col: control, RTR: rapid temperature rising, CHT: constant high temperature, RTD: rapid temperature decline and CLT constant low temperature.

**Table 3:** Primers for RT-PCR.

Gene	Sequences (5′-3′)
*AANAT*	225 bp
Forward	5-GAGCAGCGGGAGGTTTGT-3
Reverse	5-ACTTGCGGTGCACGATGGAG-3
*ASMT/HIOMT*	245 bp
Forward	5-ACAGCCTTTGACCTCTCACG-3
Reverse	5-ACCCGGGCAAGAATGAAGAG-3
*Actin*	383 bp
Forward	5-CACTATTGGCAACGAGCGGTTC-3
Reverse	5-ACTTGCGGTGCACGATGGAG-3
